# Members of WRKY Group III Transcription Factors Are Important in Mite Infestation in Strawberry (*Fragaria* × *ananassa* Duch.)

**DOI:** 10.3390/plants13192822

**Published:** 2024-10-09

**Authors:** Peng Chen, Xianhong Zhou, Haiting Wang, Xiuxia Zhang, Lei Wang, Huanhuan Gao, Qianying Zhuang, Heqin Li, Ansheng Zhang

**Affiliations:** 1 Institute of Plant Protection, Shandong Academy of Agricultural Sciences, Jinan 250100, China; cpengchina@126.com (P.C.);; 2 Shandong Key Laboratory for Green Prevention and Control of Agricultural Pests, Jinan 250100, China; 3 Key Laboratory of Natural Enemies Insects, Ministry of Agriculture and Rural Affairs, Jinan 250100, China; 4 Jining Agricultural Technology Extension Center, Jining 272000, China; 5 College of Plant Protection, Shandong Agricultural University, Tai’an 271018, China; 6 Shandong Provincial Key Laboratory of Dryland Technology, College of Agronomy, Qingdao Agricultural University, Qingdao 266109, China

**Keywords:** strawberry, WRKY transcription factors, tetranychid mite, subcellular localization, resistance

## Abstract

Strawberry is frequently attacked by mites, which directly affects the yield and quality of this fruit species. The WRKY Group III transcription factors (TFs) play an important role in plant tolerance to biotic sources of stress, such as pathogens and insect pests. In this study, six Group III WRKY TFs (*FaWRKY25*, *FaWRKY31*, *FaWRKY32*, *FaWRKY43*, *FaWRKY44*, and *FaWRKY45*) were identified in strawberry. A phylogenetic analysis showed that the six WRKY III TFs were divided into two clades and all had a conserved WRKYGQK domain and the C-X_7_-C-X_23_-H-T-C zinc finger motif. An interaction network analysis revealed that FaWRKY44 was co-expressing with FaWRKY25 and FaWRKY45. The expression patterns showed that the WRKY Group III genes responded to plant hormones and mite infestation in strawberry. To further verify the role of *FaWRKY25* in plant resistance to mites, we cloned the *FaWRKY25* gene and overexpressed it in transgenic plants. An in vivo subcellular localization analysis indicated that the FaWRKY25 protein was localized in the nucleus. Fewer mites were also detected on the wild-type plants than on *FaWRKY25*-overexpressing transgenic plants, suggesting that *FaWRKY25* negatively regulates the resistance of strawberry to mites. The present study advances our understanding on a potential target that mites use to manipulate host plant defenses.

## 1. Introduction

Cultivated strawberry (*Fragaria* × *ananassa* Duch.) is an economically highly valued fruit crop species belonging to the Rosaceae family, which also includes apple, peach, and cherry. Strawberries frequently encounter various adverse environmental conditions, such as, but not limited to, drought and salinity, as well as fungal and bacterial pathogens and pests [1]. The tetranychid mite is one of the most serious agricultural pests of strawberries worldwide and directly affects the yield and quality of the berries [2,3]. In particular, they damage the leaves, decrease the plants’ ability to photosynthesize, and cause defoliation [3]. Cultivated strawberries have evolved specific immune mechanisms to cope with different kinds of stresses [4]. The mechanisms protecting plants against insect pests are highly regulated processes. One of the vital steps in these mechanisms is the transcriptional regulation of numerous defense-related genes by various transcription factors [5]. Previous studies have revealed that WRKY transcription factors play important roles in the stress responses to various biotic factors [6,7,8].

The WRKY transcription factors (TFs) belong to a large plant-specific TF family and contain a highly conserved WRKY domain [9,10]. WRKY TFs can be classified into three main groups (Groups I, II, and III) based on the number of WRKY domains and their zinc-finger structure [11]. Among the three groups, Group III is likely the most dynamically evolving and adaptable group within the gene family [12,13,14]. In plants, the number of WRKY TFs was closely associated with the number of Group III WRKY gene family [12]. For instance, 13, 10, 28, 11, 10, and 6 Group III WRKY genes were detected in *Arabidopsis* [5], *Populus* [12], rice [12], pea [15], wild strawberry [16], and cultivated strawberry [17], respectively. The fewer number of group III WRKY gene members is a potential reason for the smaller number of WRKY family members in cultivated strawberries [17].

The WRKY Group III TFs were reported to be induced by biotic stresses (e.g., pathogens, viruses, sucking pests, etc.) and to participate in the regulation of the plant’s tolerance to these stress factors [18,19]. For instance, the majority of the WRKY Group III TF members in *Arabidopsis* responded to pathogen infection [5]. The overexpression of *OsWRKY45* in rice improved the plants’ resistance to pathogen attack [20]. Zhang et al. also found that *CmWRKY53* contributes to the susceptibility of chrysanthemum to aphids [21]. Furthermore, WRKY Group III TFs take part in certain signal transduction processes mediated by hormones, such as salicylic acid (SA), jasmonic acid (JA), and abscisic acid (ABA), all of which are vital hormones that defend plants against diseases and insect pests [22,23,24,25,26]. A previous study showed that WRKY TFs participate in the interaction between SA and JA-mediated signaling [27,28]. GhWRKY Group III gene members in cotton could be induced by SA, JA, and ABA, and AtWRKY genes in *Arabidopsis* could be induced by SA treatments [5,10].

Strawberry is a popular soft fruit that is available worldwide but is prone to various pests. Chen et al. conducted a genome-wide analysis of strawberry WRKY genes, identified 47 genes in cultivated strawberry, and categorized them into three major groups, namely, Groups I, II, and III [17]. Given that WRKY Group III TFs are involved in responses to herbivores, pathogens, and hormones, we hypothesized that they could also mediate the responses of strawberry to tetranychid mites. Our current study aimed to understand the interaction of six WRKY Group III members with agricultural pests, notably tetranychid mites, of strawberry. A comprehensive analysis of the WRKY Group III proteins, including phylogenetic relationships, conserved motifs, structural models, and an interaction network, was performed. We also analyzed the gene expression profiles in response to plant hormones and mites. In order to verify the role of FaWRKY25 in plant resistance to mites, we cloned the *FaWRKY25* in strawberry and studied its function by overexpressing it in transgenic plants. The results showed that *FaWRKY25* negatively regulates the resistance of strawberry to mites.

## 2. Results

### 2.1. Phylogenetic Analysis of the WRKY Group III Proteins in Strawberry

**Figure 1:**
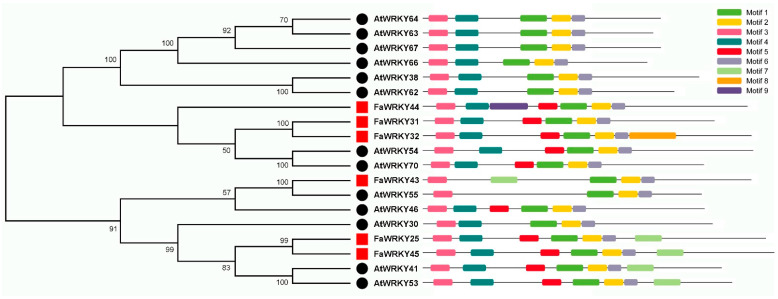
Phylogenetic tree and motif components’ map of the FaWRKY (strawberry, red squares) and AtWRKY (*Arabidopsis*, black circles) proteins. The black lines represent the non-conserved sequences. Motifs 1–9 indicate the nine conserved motifs in the group III FaWRKY and AtWRKY proteins.

**Figure 2:**
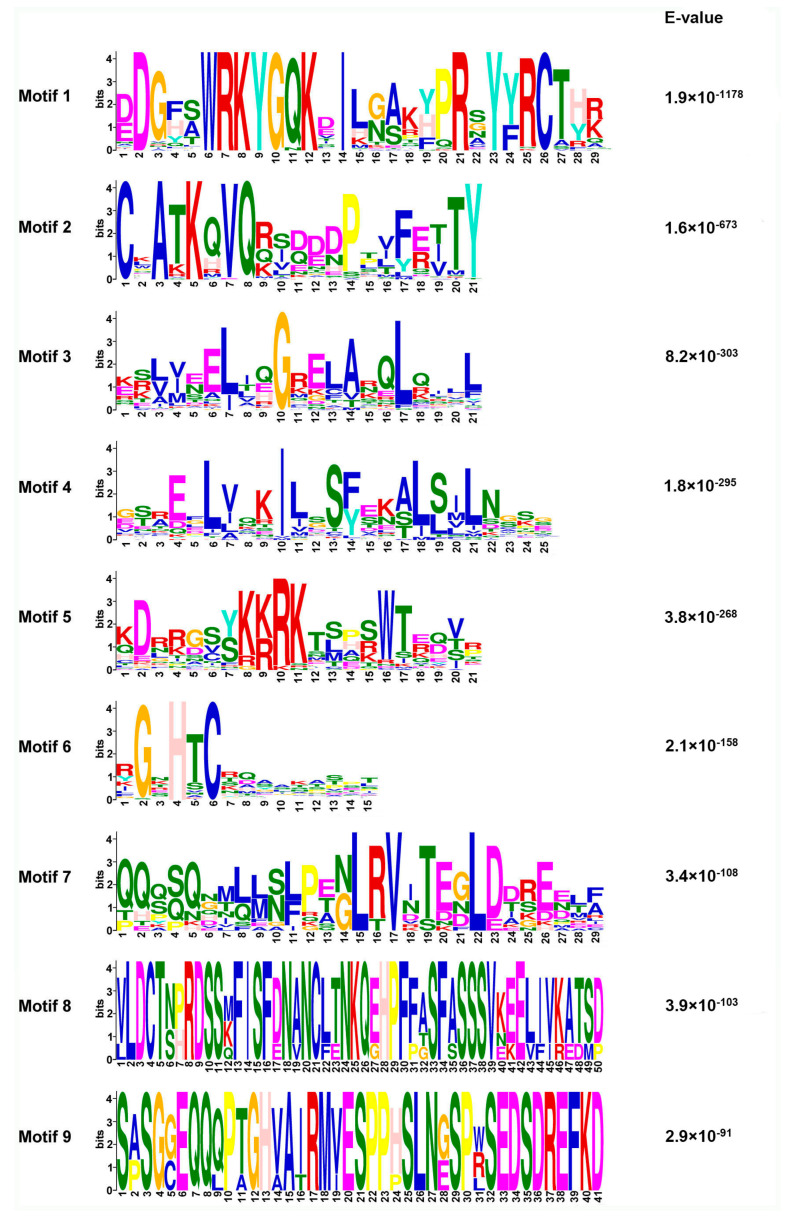
Conserved motif prediction and analysis in Group III WRKY proteins. Nine conserved motifs were identified in the WRKY group III FaWRKY and AtWRKY proteins using MEME. The conserved amino acid sequences of each motif, with larger letters designating more frequently occurring amino acids at each respective position, and relative length of each motif are shown.

Our previous study uncovered a total of six Group III WRKY proteins (FaWRKY25, FaWRKY31, FaWRKY32, FaWRKY43, FaWRKY44, and FaWRKY45) in *Fragaria* × *ananassa* Duch. Benihoppe. To explore the phylogenetic relationship of the six WRKY III genes in strawberry and in relation to *Arabidopsis*, a neighbor-joining phylogenetic tree was constructed. The results showed that the homologous proteins with similar motif components clustered together (Figure 1 and Figure 2). The WRKY III proteins were divided into two clades, one with eight motifs and the other with seven motifs. All six FaWRKY Group III TFs had a conserved WRKYGQK domain and a C-X7-C-X23-H-T-C zinc finger motif. The motifs 1, 2, and 3 were widely present in all FaWRKY (strawberry) and AtWRKY (*Arabidopsis*) proteins. All six FaWRKY Group III proteins contained motifs 1, 2, 3, and 6. With the exception of FaWRKY43, all FaWRKY Group III proteins also contained the conserved sequence motifs 4 and 5. Furthermore, two strawberry pairs (FaWRKY25 and FaWRKY45, FaWRKY31 and FaWRKY32), one strawberry and *Arabidopsis* pairs (FaWRKY43 and AtWRKY55), and four *Arabidopsis* pairs (AtWRKY64 and AtWRKY63, AtWRKY38 and AtWRKY62, AtWRKY54 and AtWRKY70, and AtWRKY41 and AtWRKY53) were identified as orthologous genes, respectively.

### 2.2. Structural Model and Interaction Network of WRKY Group III Proteins in Strawberry

**Figure 3:**
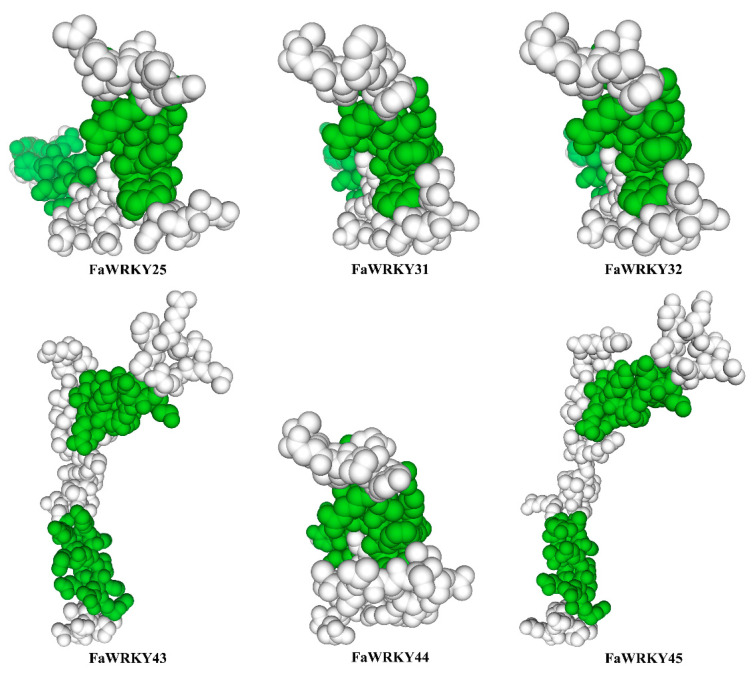
Structural models of the strawberry WRKY group III protein. The green color indicates the binding site.

The structural models of six strawberry WRKY Group III proteins were examined to identify putative binding sites. The structure of the FaWRKY31, FaWRKY32, and FaWRKY44 domains were predicted to be very similar (Figure 3). FaWRKY43 and FaWRKY45 contained two binding sites, but the other four FaWRKY domains contained one binding site (Figure 3).

**Figure 4:**
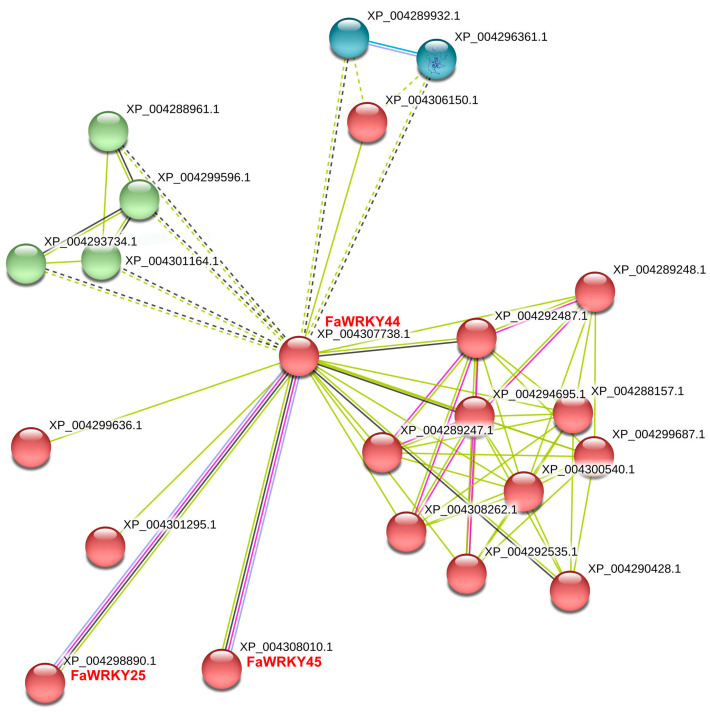
Interaction network of the strawberry WRKY III protein.

Furthermore, we analyzed the interaction network of the WRKY Group III proteins to predict their regulation mechanism. As shown in Figure 4, three Group III proteins (FaWRKY25, FaWRKY44, and FaWRKY45) interacted with other proteins in the strawberry genome. FaWRKY44 also shared co-expression patterns with both FaWRKY25 and FaWRKY45.

### 2.3. Expression Patterns of Group III FaWRKY Genes in Response to Hormone Treatments

**Figure 5:**
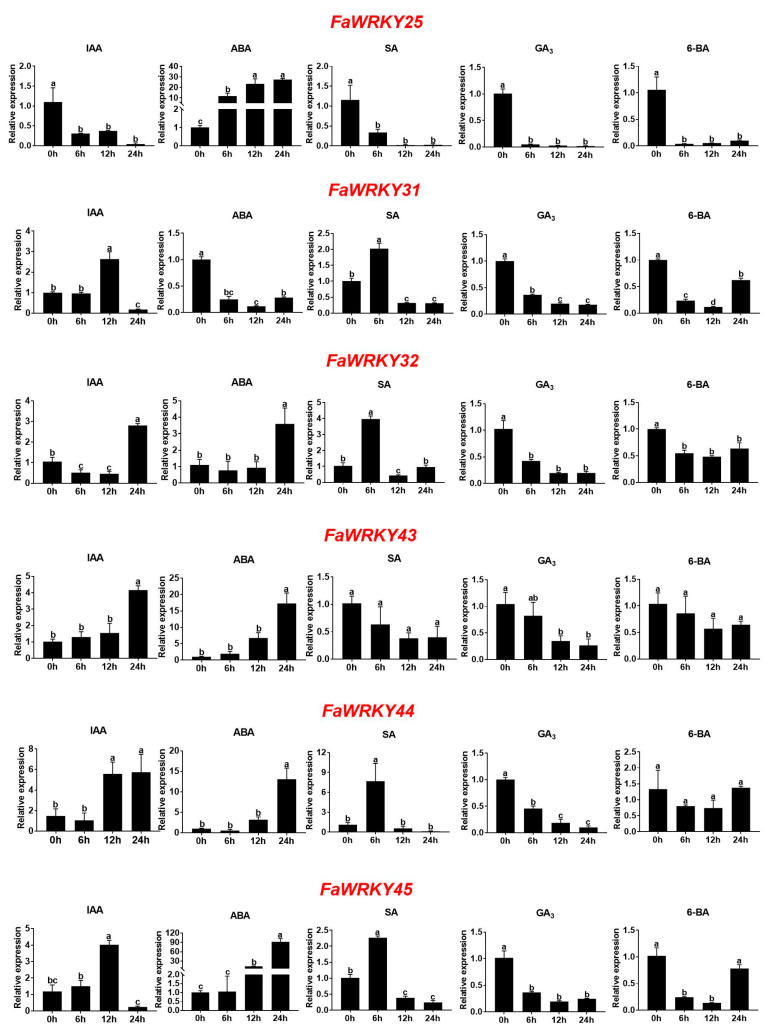
Expression patterns of six Group III FaWRKY genes in response to five hormone treatments. The letters above the values indicate significant differences in different treatments (analysis of variance [ANOVA]: Duncan test; *p*  <  0.05).

To assess the role of Group III FaWRKY genes in the response mechanism to abiotic sources of stress, such as indole-3-acetic acid (IAA), ABA, SA, gibberellin A_3_ (GA_3_), and 6-Benzylaminopurine (6-BA), strawberry leaves were treated with each of the hormones and sampled at 0 h, 6 h, 12 h, and 24 h post-treatment, with 0 h as the control. The expression patterns of the six Group III FaWRKY genes were determined using qRT-PCR (Figure 5). The results showed that FaWRKY25 was up-regulated when treated with ABA and down-regulated by the four other compounds (Figure 5). The FaWRKY31 expression displayed a unimodal trend after IAA and SA treatments and was down-regulated after ABA, GA_3_, and 6-BA treatments. The FaWRKY43 expression was up-regulated at 24 h when treated with IAA and ABA and was down-regulated at 12 h when treated with GA_3_. FaWRKY44 was up-regulated when treated with IAA (12 h and 24 h), ABA (24 h), and SA (6 h) and down-regulated when treated with GA_3_. FaWRKY32 and FaWRKY45 displayed similar expression patterns and were up-regulated when treated with SA (6 h), IAA (24 h or 12 h), and ABA (24 h) but down-regulated after GA_3_ and 6-BA treatments.

### 2.4. Expression Patterns of Six Group III FaWRKY Genes in Response to Tetranychid Mites

**Figure 6:**
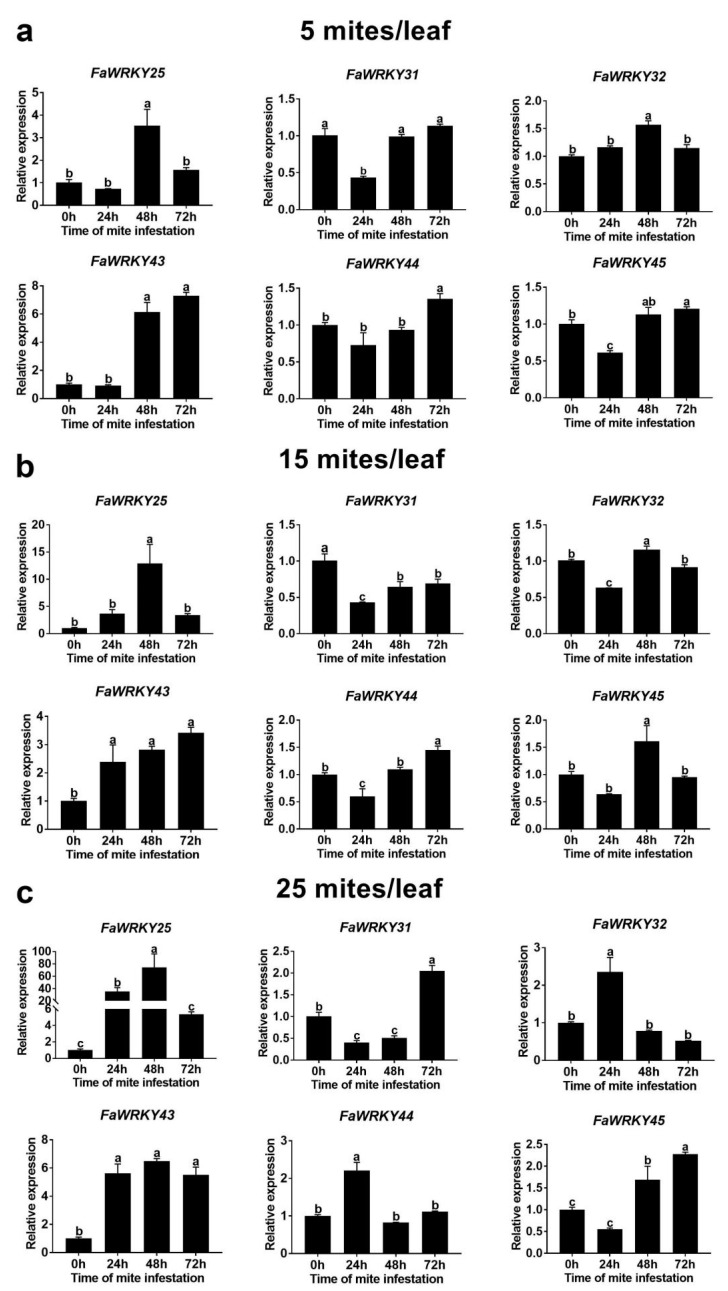
Expression patterns of six Group III FaWRKY genes in response to different densities of *Tetranychus urticae* Koch infestation (5, 15, and 25 mites/leaf). The letters above the values indicate significant differences in different treatments (analysis of variance [ANOVA]: Duncan test; *p*  <  0.05).

To further explore the possible role of the Group III FaWRKY genes after tetranychid mite infection at different time points, the expression levels of the six FaWRKY genes belonging to the Group III subfamily were analyzed using qRT-PCR (Figure 6). The results showed that a mite density of five mites/leaf significantly increased the FaWRKY25 and FaWRKY32 expression levels at 48 h, the FaWRKY44 and FaWRKY45 expression levels at 72 h, and those of FaWRKY43 at both 48 h and 72 h (Figure 6). However, the expression level of FaWRKY31 was significantly inhibited in the infested leaves at 24 h. A mite density of 15 mites/leaf significantly increased the FaWRKY25 and FaWRKY45 expression levels at 48 h and resulted in a decreasing and then increasing trend in those of FaWRKY32 and FaWRKY44 and monotonically increasing and decreasing trends in FaWRKY43 and FaWRKY31, respectively (Figure 6). When the mite density was 25 mites/leaf, the FaWRKY25 expression levels significantly increased at 24 h and 48 h, and those of FaWRKY31 showed a decreasing and then increasing trend, while FaWRKY32 and FaWRKY44 only became up-regulated at 24 h, and FaWRKY43 and FaWRKY45 showed monotonically increasing trends (Figure 6).

### 2.5. Sequence Characteristics and Subcellular Localization of FaWRKY25

**Figure 7:**
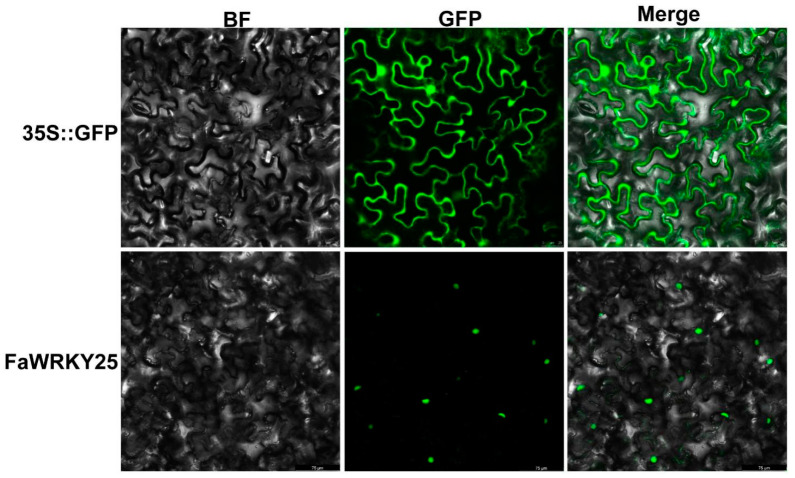
Subcellular localization of FaWRKY25. BF: Image after bright field. GFP: Image after green fluorescence. Merge represents the image after merger of bright field and green fluorescence.

The expression levels of FaWRKY25 were significantly upregulated in response to mite infestation, inferring that FaWRKY25 may be involved in the resistance of strawberry to mites. To test this hypothesis, we cloned the *FaWRKY25* gene from the strawberry hybrid *Fragaria* × *ananassa* Duch. Benihoppe. The sequence of *FaWRKY25* (PQ212725) contained a 1068 bp open reading frame encoding a polypeptide of 355 amino acid residues. To determine the subcellular localization of FaWRKY25, the FaWRKY25-GFP constructs and empty vectors containing 35S::GFP were transformed into the leaves of *N. benthamiana*. A confocal laser scanning microscopy showed that the FaWRKY25-GFP signal was clearly visible only in the nucleus of the *N. benthamiana* leaf cells, while GFP fluorescence was evenly distributed throughout the cells containing 35S::GFP (Figure 7). These in vivo results provided evidence that FaWRKY25 are nuclear proteins.

### 2.6. FaWRKY25 Contributes to the Mites’ Susceptibility to Strawberry

**Figure 8:**
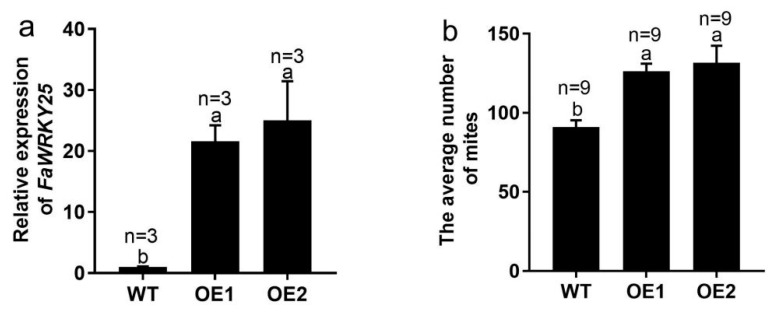
Identification of FaWRKY25 transgenic plants and the mite population size on FaWRKY25-overexpressing plants. (**a**) Relative expression levels of FaWRKY25 in the wild-type (WT) and transgenic (OE1 and OE2) plants, the columns and error bars represent means ± SE (n = 3). (**b**) The average number of mites on the wild-type and transgenic lines, the columns and error bars represent means ± SE (n = 9). The letters above the values indicate significant differences in different treatments (analysis of variance [ANOVA]: Duncan test; *p*  <  0.05).

To determine the function of FaWRKY53, transgenic strawberry lines that were overexpressing the gene were obtained. The transgenic plants were verified using qRT-PCR (Figure 8a). The infestation assays of the tetranychid mites showed that the number of mites on the wild-type plants was lower than on the FaWRKY25-overexpressing transgenic plants (Figure 8b). The mites’ multiplication rate (MR) on the wild-type plants (4.55) was also lower than on the FaWRKY25-overexpressing lines (6.32 and 5.01). The inhibition ratio (IR) for OE1 and OE2 lines were −38.83% and −44.69%, respectively (Table 1), suggesting that FaWRKY25 contributed to increasing the sensitivity of strawberry to mites.

## 3. Discussion

### 3.1. Structural Characteristics of FaWRKY Group III TFs

The WRKY Group III transcription factors are a class of DNA-binding proteins, most of which are involved in the regulation of plant innate immunity and respond to a wide range of stressors [17,29,30]. The WRKY Group III TFs mainly contain one WRKYGQK domain in the N-terminus and a C_2_HC (C-X_7_-C-X_23_-H-X-C) zinc finger motif [11,31]. In this study, all six FaWRKY Group III TFs conform to these characteristics. The WRKY protein functions via interactions with a diverse array of protein partners, such as other WRKY transcription factors [32]. In this study, three proteins (FaWRKY44, FaWRKY25, and FaWRKY45) with significant structural differences interacted with each other within the network. Similar phenomena were reported in the tomato genome, where two Group III proteins SolyWRKY80 and SolyWRKY53 were significantly correlated with SolyWRKY17 [33]. In the mites’ defense, the FaWRKY Group III TFs might serve as regulators in the strawberry defense network and assist in response to mites.

### 3.2. FaWRKY Group III Genes Involved in Hormone-Mediated Pathways

Previous studies showed that WRKY TFs regulate hormone-mediated signaling pathways, such as the PsWRKY TFs that can respond to ABA, IAA, GA, MeJA, and SA [15]. An analysis of the stress-related cis-acting elements revealed that most FaWRKY promoter regions had one or more hormone-responsive elements [17]. In this study, we examined the expression levels of the six FaWRKY genes after plants were treated with each of five hormones (IAA, ABA, SA, GA_3_, and 6-BA). We found that the gene expression of some of these genes showed a monotonically increasing or decreasing trend, whereas others showed a unimodal trend in response to specific hormones. Many WRKY genes were reported to play vital roles in regulating the ABA-mediated pathway; for instance, SlWRKY6 enhanced drought tolerance via the ABA-dependent pathway in *Solanum lycopersicum* L. [34]. In our study, five FaWRKY Group III genes, including FaWRKY25, FaWRKY32, FaWRKY43, FaWRKY44, and FaWRKY45, were also shown to be activated by exogenous ABA at different times. These observations suggest that most of the FaWRKY Group III genes play important roles in the ABA-dependent pathway. Moreover, the SA signaling pathways were reported to contribute to plant defense, and some Group III WRKY TFs were shown to be induced by SA [15]. FaWRKY31, FaWRKY32, FaWRKY44, and FaWRKY45 in strawberry were also activated by SA, suggesting that the FaWRKY Group III genes may be involved in plant immunity. Moreover, AtWRKY70 was shown to promote the expression of SA-induced genes in Arabidopsis, and CsNPR1 was shown to recruit CsWRKY11 to amplify the primary SA signal in cucumber [35,36].

### 3.3. Expression Profiles of FaWRKY Group III Genes in Response to Mites

In this study, the expression levels of six FaWRKY Group III genes (FaWRKY25, FaWRKY31, FaWRKY32, FaWRKY43, FaWRKY44, and FaWRKY45) were detected at different time points after the plants were infested with mites (Figure 6). The FaWRKY Group III genes may be involved in the plant’s ability to resist mite infestation by inducing corresponding expression patterns in strawberry. Overexpressing WRKY genes have been reported to significantly help regulate plant defense mechanisms against certain biotic sources of stress [37,38]. In tobacco, overexpressing GhWRKY25 enhanced the plant’s sensitivity to fungal pathogens by inducing the SA/JA signaling pathway [39]. In tomato, Group III genes served as positive (SolyWRKY41 and SolyWRKY54) and negative (SolyWRKY42 and SolyWRKY80) regulators when interacting with TYLCV [33]. In our study, five FaWRKY Group III genes (FaWRKY25, FaWRKY32, FaWRKY43, FaWRKY44, and FaWRKY45) were differentially activated by different numbers of mites, while FaWRKY31 was downregulated. These results were highly consistent with the previous results from ABA treatment. Therefore, the ABA content might be enhanced by mite infestation, and then ABA might contribute to the expression of FaWRKY Group III genes.

### 3.4. FaWRKY25 Negatively Regulates the Resistance of Strawberry to Mites

Numerous studies shpowed that the WRKY genes are involved in pest resistance [21]. Upregulating AtWRKY22 was shown to render *Arabidopsis* susceptible to *Myzus persicae* by modulating the plant’s SA and JA signaling pathways [8]. In chrysanthemum, *CmWRKY53* contributed to the increased sensitivity of the plant to aphids by decreasing the levels of secondary metabolites [21]. In the present study, similar to *CmWRKY53*, *FaWRKY25* was shown to contribute to increasing the sensitivity of strawberry to mites.

## 4. Materials and Methods

### 4.1. Data Collection

The Group III TFs were obtained from Strawberry GARDEN (http://strawberry-garden.kazusa.or.jp, accessed on 10 June 2018) [40]. The *Arabidopsis thaliana* WRKY Group III member sequences were retrieved from the National Center for Biotechnology Information (NCBI, https://www.ncbi.nlm.nih.gov/).

### 4.2. Sequence Analysis

Multiple alignments of the characterized WRKY Group III proteins were performed using Clustal X (v1.83). The neighbor-joining (NJ) phylogenetic tree with 1000 bootstraps was constructed using MEGA 7.0 [41,42]. Conserved motifs of WRKY Group III protein sequences were identified using the MEME Suite(https://meme-suite.org/meme/, accessed on 9 December 2019) [43]. The SWISS-MODEL (https://swissmodel.expasy.org, accessed on 23 July 2019) for protein sequence analysis was used to identify structural models in the strawberry WRKY Group III proteins [44]. The interaction network of WRKY Group III was analyzed by SMART(http://smart.embl.de/, accessed on 9 December 2019).

### 4.3. Plant Materials, Growth Conditions, and Stress Assays

*Fragaria* × *ananassa* Duch. Benihoppe were planted in pots and grown at 22 ± 1 °C in a greenhouse with a 16 h light/8 h dark photoperiod. Healthy strawberries with the same leaf size and number of leaves were selected and inoculated with the female adult *Tetranychus urticae* Koch.

For the plant hormone stress experiment, IAA, ABA, SA, GA_3_, and 6-BA solutions at a concentration of 100 mM were prepared and sprayed evenly on the strawberry leaves. The control plants were sprayed with water. The samples were then collected at 0 h (CK), 6 h, 12 h, and 24 h after spraying. For the biotic stress experiment, the mite population densities were 5, 15, and 25 mites/leaf, respectively. Petroleum jelly was applied to the petioles to prevent the escape of mites. Leaves were collected at 0 h (CK), 24 h, 48 h, and 72 h after mite infestation. Each sample included three replicates, and each replicate contained the leaves from three plants. All of the collected leaf samples were snap-frozen in liquid nitrogen and stored at −80 °C.

### 4.4. RNA Extraction and Quantitative Real-Time PCR Analysis

The total RNA was isolated from the strawberry leaves using the plant RNA Kit (BioTeke, Beijing, China) in accordance with the manufacturer’s protocols. The 250 ng of total RNA was reverse transcribed to the first-strand cDNA using the FastQuant RT Kit (TIANGEN, Beijing, China). The qRT-PCR primers for the FaWRKY Group III gene members were obtained from qPrimerDB (https://biodb.swu.edu.cn/qprimerdb/; Table S1) [17,45]. The qRT-PCR reaction [46] was performed using a QuantStudio™ 6 Flex Real-Time PCR System (Applied Biosystems, Thermo Fisher Scientifc, Waltham, MA, USA). The 20 μL reaction for each sample contained 10 μL of 2× SYBR^®^ Select Master Mix (Applied Biosystems, Thermo Fisher Scientifc, Waltham, MA, USA), 1 μL of diluted cDNA, 1 μL of each of two primers, and 7 μL of RNase-free water. Each sample reaction was carried out in three independent biological and technical replicates. The housekeeping DNA binding protein gene (EU727547) was used as an internal control [46]. The relative gene expression level was calculated using the 2^−ΔΔCT^ method [47].

### 4.5. Isolation and Subcellular Localization of FaWRKY25

The total RNA was extracted from the strawberry leaves using the plant RNA Kit (BioTeke, Beijing, China). The FaWRKY25-F/R primers were used in PCR to amplify the FaWRKY25 open reading frame (ORF; Table S2). The PCR product was purified and cloned into pMD19-T (TaKaRa, Otsu, Shiga, Japan) for sequencing.

The ORF of FaWRKY25 without stop codons was cloned into a pCAMBIA1302 overexpression vector, which carries the green fluorescent protein (GFP). The empty pCAMBIA1302 vector containing the 35S::GFP fusion protein was used as the control (Table S2). The constructed plasmids were individually introduced into *Agrobacterium tumefaciens* EHA105 and were then transferred into *Nicotiana benthamiana* leaves. The transient expression of GFP was observed using a Zeiss LSM800 (Oberkochen, Baden-Württemberg, Germany) laser scanning confocal microscope.

### 4.6. Generation of FaWRKY25-Overexpressing Transgenic Plants

The FaWRKY25 ORF with the stop codons was cloned into a pCAMBIA2301 overexpression vector. The recombinant plasmid was transformed into *A. tumefaciens* EHA105 and subsequently introduced into strawberry via *Agrobacterium*-mediated transformation (Figure S1) [48]. Transgenic plants were identified by PCR, and the expression levels of FaWRKY25 were measured using qRT-PCR. The primer pairs used are listed in Tables S1 and S2.

### 4.7. Analysis of Mite Resistance in FaWRKY25-Overexpressing Transgenic Plants

To assess mite population size, 4–6-leaf stage wildtype and FaWRKY25-overexpressing transgenic strawberry plants were infested with 20 recently hatched mites. The total number of mites per plant was counted at 7 days after infestation. The multiplication rate (MR = Ni/20, where Ni represents the total number of mites on each plant) and inhibition ratio (IR = 100 (NW − NT)/NW, where NW and NT represent the mean numbers of mites counted at 7 days after infestation on wild-type and transgenic plants) were used to quantify the degree of plant resistance [21,49]. Each assay contained three biological replicates, and each biological replicate included three plants from each line.

### 4.8. Statistical Analyses

Statistical analyses were conducted using *t*-test with SPSS 20.0 (SPSS Inc., Armonk, NY, USA). Figures were created using GraphPad Prism 7.0 (La Jolla, CA, USA).

## 5. Conclusions

In this study, six WRKY Group III genes in strawberry were examined, and the phylogenetic relationship analysis showed that the TFs fall into two clades. An interaction network analysis showed that FaWRKY44 was co-expressed with FaWRKY25 and FaWRKY45. The expression levels of the six FaWRKY Group III genes changed significantly in response to five hormones and different mite infestation densities. Furthermore, the strawberry FaWRKY25 was shown to localize in the plant cell nucleus when cloned and transformed into *N. benthamiana* leaf cells, and the number of mites decreased in FaWRKY25-overexpressing plants. These results showed that the increased expression level of *FaWRKY25* mediates the strawberry’s susceptibility to mites. Our study provides a theoretical basis on the function and mechanism of WRKY Group III genes, especially *FaWRKY25*, with mites on strawberry.

## Figures and Tables

**Table 1:** Multiplication rate (MR) and inhibition ratio (IR) of mites in the wild-type (WT) and FaWRKY25 transgenic lines (OE1 and OE2) at 7 days after infestation.

	WT	OE1	OE2
MR	4.55	6.32	5.01
IR (%)	0.00	−38.83	−44.69

## Data Availability

The data are contained within this article and Supplementary Materials.

## Supplementary Materials

The following supporting information can be downloaded at: https://www.mdpi.com/article/10.3390/plants13192822/s1, Table S1: Primers of q-PCR; Table S2: Primer sequence of FaWRKY25; and Figure S1: Acquisition of FaWRKY25-overexpressing transgenic plants.
