# Supplementary material for: Members of WRKY Group III Transcription Factors Are Important in Mite Infestation in Strawberry (Fragaria × ananassa Duch.)
Source: Plants (Basel). 2024 Oct 9;13(19):2822. doi: 10.3390/plants13192822 (PMC11478921; doi:10.3390/plants13192822)
Supplement: Supplementary file 1 [file plants-13-02822-s001.zip › Table S1-2.pdf]

Table S1 Primers of q-PCR

Gene name		Sequences 5'→3'	Annealing Temperature
FaWRKY25	Forward	CCTTTAGGAACACACAAAGCTG	58℃
	Reverse	TCTTCCAAATTCTCAGTGTCGA	
FaWRKY31	Forward	ATAACGTAATCGGTGGTAGTCC	58℃
	Reverse	AAGGGGTGTTCTAGTTCGTAAG	
FaWRKY32	Forward	GTACCGAAACGTTTAAGGATCG	58℃
	Reverse	AGAATCGTCTTTTGTCCGTACT	
FaWRKY43	Forward	GAGGATCATTACATCCAAACGC	58℃
	Reverse	ACGGTCAATTTACGAATTTCCC	
FaWRKY44	Forward	ATTGTCGATGATTCCCTTCCTT	58℃
	Reverse	GCATGTTCGAAACCACATTTTC	
FaWRKY45	Forward	AGGACATTCTTGGAGCCAAATA	58℃
	Reverse	TGCCTTCCTCGGTAAGTAATTT	

Table S2 Primer sequence of FaWRKY25

Gene name		Sequences 5'→3'	Annealing Temperature
FaWRKY25 (clone)	Forward	ATGGATACTGCTAGTAAGAGCTG	56 °C
	Reverse	CCTGAAACCTAAGTAGAATTATCAG	
FaWRKY25 (pCAMBIA1302)	Forward	CCCATGGGCCATGGATACTGCTAGTAAGAGCTGGG	61°C
	Reverse	GGACTAGTCCGAAAAATCCTGGAGTATCAAATGGG	
FaWRKY25 (pCAMBIA2301)	Forward	CCCCGGGGATGGATACTGCTAGTAAGAGCTG	55 °C
	Reverse	CGGATCCGCCTGAAACCTAAGTAGAATTATCAG	
