# Supplementary figures and images for: Members of WRKY Group III Transcription Factors Are Important in Mite Infestation in Strawberry (Fragaria × ananassa Duch.)
Source: Plants (Basel). 2024 Oct 9;13(19):2822. doi: 10.3390/plants13192822 (PMC11478921; doi:10.3390/plants13192822)

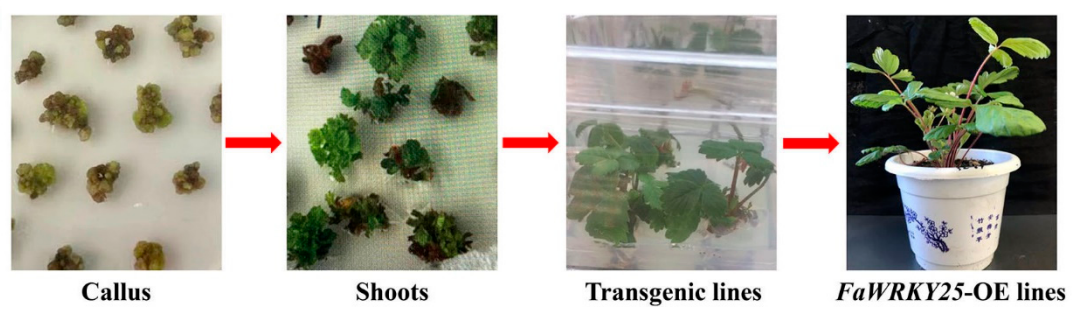

Figure S1 Acquisition of *FaWRKY25*-overexpressing transgenic plants.

Supplement: Supplementary file 1 [file plants-13-02822-s001.zip › Fig.S1.pdf]
